# Hyperoxidation of Peroxiredoxin 6 Induces Alteration from Dimeric to Oligomeric State

**DOI:** 10.3390/antiox8020033

**Published:** 2019-02-02

**Authors:** Sharifun Shahnaj, Rimpy Kaur Chowhan, Potshangbam Angamba Meetei, Pushpa Kakchingtabam, Khundrakpam Herojit Singh, Laishram Rajendrakumar Singh, Potshangbam Nongdam, Aron B. Fisher, Hamidur Rahaman

**Affiliations:** 1 Department of Biotechnology, Manipur University, Manipur 795003, India; sharifun15@gmail.com (S.S.); angambameetei@gmail.com (P.A.M.); pushpakakchingtabam@gmail.com (P.K.); herojit009@gmail.com (K.H.S.); purenba@rediffmail.com (P.N.); 2 ACBR, Delhi University, New Delhi, Delhi 110022, India; rimpy_1989@yahoo.co.in (R.K.C.); lairksingh@gmail.com (L.R.S.); 3 Institute for Environmental Medicine, University of Pennsylvania Perelman School of Medicine, Philadelphia, PA 19104-6068, USA; abf@upenn.edu

**Keywords:** peroxidatic cysteine, thioredoxin fold, sulfonic/sulfinic acid, phospholipase A2 activity, reactive oxygen species

## Abstract

Peroxiredoxins(Prdx), the family of non-selenium glutathione peroxidases, are important antioxidant enzymes that defend our system from the toxic reactive oxygen species (ROS). They are thiol-based peroxidases that utilize self-oxidation of their peroxidatic cysteine (C_p_) group to reduce peroxides and peroxidized biomolecules. However, because of its high affinity for hydrogen peroxide this peroxidatic cysteine moiety is extremely susceptible to hyperoxidation, forming peroxidase inactive sulfinic acid (Cys-SO_2_H) and sulfonic acid (Cys-SO_3_H) derivatives. With the exception of peroxiredoxin 6 (Prdx6), hyperoxidized sulfinic forms of Prdx can be reversed to restore peroxidase activity by the ATP-dependent enzyme sulfiredoxin. Interestingly, hyperoxidized Prdx6 protein seems to have physiological significance as hyperoxidation has been reported to dramatically upregulate its calcium independent phospholipase A_2_ activity. Using biochemical studies and molecular dynamic (MD) simulation, we investigated the roles of thermodynamic, structural and internal flexibility of Prdx6 to comprehend the structural alteration of the protein in the oxidized state. We observed the loosening of the hydrophobic core of the enzyme in its secondary and tertiary structures. These changes do not affect the internal dynamics of the protein (as indicated by root-mean-square deviation, RMSD and root mean square fluctuation, RMSF plots). Native-PAGE and dynamic light scattering experiments revealed the formation of higher oligomers of Prdx6 under hyperoxidation. Our study demonstrates that post translational modification (like hyperoxidation) in Prdx6 can result in major alterations of its multimeric status.

## 1. Introduction

Reactive oxygen species (ROS) are generated as a result of normal cellular metabolism such as aerobic respiration, photosynthesis, and exposure to environmental stimuli [1]. Depending on their concentration in biological systems, ROS can either be harmful or beneficial to living systems [2]. Prolonged presence of these imbalanced reactive oxygen intermediates further initiates a chain reaction where any macromolecule coming into contact with them is oxygenated, thereby exponentially enhancing the ROS concentration and cellular damage. In certain cases, it is very difficult to distinguish whether oxidative stress is the cause or an effect of a disease.

To combat the exponential growth of ROS, humans are equipped with a robust antioxidant defense system, comprising enzymatic and non-enzymatic antioxidants that work synergistically and interactively to neutralize free radicals. These antioxidants can be both endogenous (reduced glutathione (GSH), superoxide dismutase, peroxiredoxins, etc.), or exogenous (tocopherol, ascorbic acid, flavonoids, etc.) in origin [3,4,5].

Among all the antioxidant enzymes found in living systems, peroxiredoxins (Prdx) are the most important thiol-dependent selenium and heme-free peroxidases and are ubiquitously found in bacteria, archaea and eukarya domains [1,2,3,6,7]. The high abundance of Prdxs in a wide range of cells and their high catalytic efficiencies in comparison to other peroxidases mean the proteins are responsible for a reduction of 90% of cellular peroxides such as hydrogen peroxide, peroxinitrite and hydroperoxides [1,2,3,6,7,8]. Because of their ROS restricting behavior, Prdxs have been implicated as a mediator in various signaling pathways involved in antioxidant-system regulation, cell proliferation, differentiation, and apoptosis in eukaryotic cells [9]. 

All Prdxs have a conserved thioredoxin domain at the N-terminal followed by C-terminal residues. These Prdxs belong to thioredoxin (Trx) fold proteins that share a common active site motif with two reactive cysteine residues; CXXC, where X can be any other amino acid and C are cysteines residues involved in the catalytic mechanism [10] and their role is to reduce disulfides. The reduced form of some Trx fold proteins contains two free thiol groups at the cysteine residues, whereas the oxidized form contains a disulfide bond between them. In contrast to Trxs, the role of Prdx is to reduce peroxides. However, similar to Trx, Prdxs have a conserved redox-active cysteine residue (the so-called peroxidatic cysteine) (CpSH) but the second catalytic cysteine is present in a different part of the protein. Mammalian Prdxs have been sub-categorized into six isoforms depending on the number of cysteine residues and their mode of involvement in the peroxidatic catalytic mechanism; typical 2-Cys Prdx (Prdx1–4 in the other nomenclature), atypical 2-Cys Prdx (Prdx5) and 1-Cys Prdx (Prdx6) [7,9,11]. Peroxiredoxin’s catalytic cycle involves three major steps [1]. The first step is peroxidation i.e., reduction of the hydroperoxide substrate leading to oxidation of N-terminal conserved Cys residue of Prdx’s to sulfenic acid (C-SOH), which could further oxidize to sulfinic acid (C-SO_2_H) and sulfonic acid (C-SO_3_H). While, C-SO_3_H is an irreversible oxidized form that causes enzyme inhibition, the C-SO_2_H of typical 2-Cys Prdx could be reduced with the aid of ATP driven sulfiredoxin catalyzed reaction into C-SOH. The second step is the resolution which involves the reduction of the sulfenic cys (C-SOH) while the third one is recycling i.e., the regeneration of the reduced cys sufhydryl (C-SH) of the active enzyme.

Typical 2-Cys Prdx have a conserved N-terminal (peroxidatic) and C-terminal (resolving) Cys residues that exist in different subunits in the obligate homodimer. Both Cys residues are involved in the peroxidase catalytic activity. Atypical 2-Cys Prdx only has one conserved N-terminal Cys residue and requires one additional but less conserved Cys residue in the same polypeptide [9]. As the name suggests, 1-Cys Prdx requires only one N-terminal conserved Cys residue for catalysis. However, to accomplish peroxidase activity with just one thiol group, 1-Cys Prdx forms a mixed disulfide with π-glutathione-s-transferase (πGST) which helps it to be glutathionylated, and later regenerated via the conjugated glutathione [12].

Peroxiredoxin 6 (Prdx6), the sole member of the mammalian 1-Cys Prdx subfamily, exists mostly as a dimer, which is aligned so that the two monomers are related not by a disulfide bond but by the hydrogen bonding network formed between two beta-strands of each monomer [13]. Also, it is a multifunctional enzyme having (i) peroxidase activity with H_2_O_2_, short chain hydroperoxides, and phospholipid hydroperoxides as substrates; (ii) calcium independent phospholipase A2 (aiPLA2) activity; and (iii) lysophosphatidylcholine acyl transferase (LPCAT) activity [14]. Interestingly, the dual functions of Prdx6 work in a mutually exclusive fashion at different pH such that at pH 7.4 (cytosol), it binds only with oxidized phospholipids (supporting its peroxidase activity) while at pH 4.0 (lysosome), it shows substrate specificity for both reduced and oxidized phospholipids (aiPLA_2_ activity) [15]. This pH mediated regulation of the Prdx6 functional switching allows smooth functioning of both activities without hampering any other biological process. As Prdx6 has affinity for oxidized substrate both at acidic and neutral pH, it shows aiPLA2 activity at both pH to play an important role in the repair of peroxidized cell membranes [16,17,18]. 

Like 2-cys Prdx, the conserved peroxidatic catalytic residue Cys-47 of Prdx6 is also prone to hyperoxidation forming hyperoxidized forms, Cys–SO_2_H and Cys–SO_3_H [19]. It should be noted that hyperoxidized Prdx6 is not a substrate for sulfiredoxin and therefore cannot be regenerated like other Prdxs, even from the Cys–SO_2_H form, making hyperoxidation irreversible and functional inactivation for Prdx6. Interestingly, it is already reported that H_2_O_2_-mediated hyperoxidation of Prdx6 has been shown to induce cell cycle arrest at the G2/M transition through up-regulation of its aiPLA_2_ activity though the hyperoxidized forms do not have peroxidase activity [19]. This effect of hyperoxidation on PLA2 activity is confirmed in a report by Zhou et.al. Our aim is to comprehend the structural alterations that incur due to hyperoxidation of Prdx6’s peroxidatic cysteine residue. These structural changes could be the mechanism that allows Prdx6 to up-regulate its aiPLA2 activity by accepting reduced phospholipid as its substrate within the cytosol. Until now, to our best knowledge, only a limited number of studies have been conducted to comparatively analyze the hyperoxidized and reduced forms of Prdx6. Here, we used molecular dynamic (MD) simulations along with various spectroscopic methods to investigate the molecular, conformational and thermodynamic studies of the different oxidation states of rat Prdx6. We found that hyperoxidation of Cys47 of Prdx6 induces changes at secondary, and tertiary as well as quaternary levels in the structure of enzyme, which might be responsible for the upregulation of its total cellular aiPLA_2_ activity. For the first time, we are reporting the existence of an oligomeric form of Prdx6 enzyme.

## 2. Materials and Methods

### 2.1. Materials

Analytical grade Tris-HCl, Dithiothreitol (DTT), and IPTG samples were purchased from the Sigma-Aldrich Corp. (St. Louis, MO, USA). H_2_O_2_ was obtained from MP Biomedicals (Santa Ana, CA, USA). NaCl was from Merck (Kenilwort, NJ, USA). This and other chemicals were analytical grade reagents and were used without further purification. 8-Anilinonaphthalene-1-sulfonate (ANS) was also purchased from the Sigma-Aldrich Corp. (St. Louis, MO, USA). Chitin resin beads were purchased from New England Biolabs (NEB) (Ipswich, MA, USA).

### 2.2. Preparation of Recombinant Prdx6

**Figure 1:**
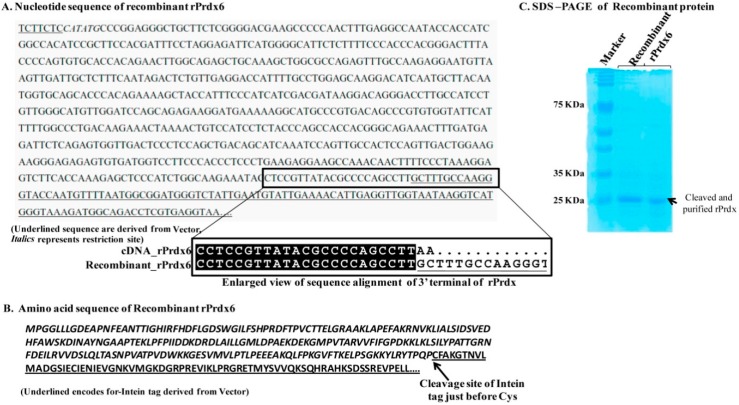
Details of nucleotide sequence of plasmid expressing recombinant rat peroxiredoxin 6 (rPrdx6). The 5′ terminal underlined and italics indicate the derived primer that was used. The 3′ terminal sequence is vector derived nucleotides after recombinant cloning. Inset view shows the different nucleotides of recombinant plasmid to cDNA sequence of rPrdx6 at 3′ terminal (**A**). The amino acid sequence of the prepared construct contains an intein tag for purification. Underlined sequence encodes for the vector derived amino acids. The cleavage site of the protein from purification tag is shown with an arrow (**B**). Sodium dodecyl sulfate polyacrylamide gel electrophoresis (SDS-PAGE) of purified Prdx6 using chitin affinity chromatography. The purity is more than 90% (**C**).

The cloning of the target gene of rat Prdx6 into the pTyB1 vector was done as described previously [20,21]. The construct contained Nde1 (at 5′ end) and sap1 (at 3′ end) as restriction sites. The restriction site of Sap1 is not generated after recombinant cloning. Thus, the nucleotide sequence of rPrdx6 is immediately followed by that of an intein tag derived from pTyB1 vector (Figure 1A,B). The recombinant plasmid was constructed to over-express the protein conjugated with an intein tag. During the purification, the protein was eluted by cleaving the intein tag, the cleavage occurred between the last residue of rPrdx and the Cys residue of the intein tag. The purified protein consists of 224 amino acids, as in the cDNA, without any vector derived residues. The recombinant vector was transformed into the *E. coli* BL21 (DE3) expression strain. A single colony of transformant was selected and inoculated in luria bertani (LB) medium containing ampicillin (50 µg/mL). Cells were grown at 37 °C in a shaker incubator overnight. When the optical density (at 600 nm) of the growing cells reached 0.6–0.8, isopropyl-β-d-thiogalactopyranoside (0.6 mM) was added for its induction. Then, the cells were grown at 20 °C overnight. After harvesting by centrifugation, the cell pellet was suspended in lysis buffer (20 mM Tris-HCl pH 7.5, 500 mM NaCl and 1 mM Ethylenediaminetetraacetic acid (EDTA)) and sonicated on ice (Bandelin sonicator, Bandelin, Germany) with five pulses of 10 s at an interval of 5 min. The lysate after sonication was centrifuged at 10,000 rpm for 20 min. The supernatant obtained after centrifugation was loaded onto a chitin affinity column equilibrated with 20 mM Tris-HCl pH 7.5, 500 mM NaCl and 1 mM EDTA. Induction of the on-column cleavage was performed by quickly flushing the column with cleavage buffer containing 20 mM Tris-HCl pH 8.5, 500 mM NaCl in presence of 80 mM DTT. After 48 h incubation of the inducted column at room temperature, the target protein was eluted with column buffer (20 mM Tris-HCl pH 7.5, 500 mM NaCl and 1 mM EDTA). The purified protein was analyzed by running 10% sodium dodecyl sulfate-polyacrylamide gel electrophoresis (SDS-PAGE) and found to be more than 90% pure as shown in Figure 1C.

### 2.3. Preparation of Reduced and Hyperoxidized Prdx6

The purified protein was used for preparation of reduced Prdx6 by adding 1 mM DTT in standard buffer i.e., 50 mM Tris-HCl, 100 mM NaCl pH 7.4. We incubated the protein with 500 µM H_2_O_2_ to get the hyperoxidized Prdx6 (C47 sulfinic acid or sulfonic acid) at room temperature for 30 min to 1 h in the standard buffer. At this concentration of H_2_O_2_ the antibody against the oxidized protein detects both the sulfinic (–SO_2_H) and sulfonic (–SO_3_H) states of the protein [15,19].

### 2.4. Circular Dichroism (CD) Measurements

CD measurements of reduced Prdx6 and hyperoxidized Prdx6 were recorded in 50 mM Tris-HCl, 100 mM NaCl, pH 7.4 using a Jasco-750 CD Spectropolarimeter. All the measurements were done at 25 °C in a thermoelectric cell holder, with the temperature being maintained using a Peltier element. Spectra were recorded in the far-ultraviolet region (190–260 nm), with a bandwidth of 1.0 nm, a step size of 1 nm, an integration time of 30 s, and with three repeats. A fused quartz cell with a pathlength of 0.1 cm was used. The protein concentration was 10 µM. The results of all the CD measurements are expressed as mean residue ellipticity ([*θ*]_λ_) in deg cm^2^ dmol^−1^ at a given wavelength λ (nm) using the relation: [*θ*]_λ_ = *θ*_λ_M_o_/10*cl*, where *θ*_λ_ is the observed ellipticity in millidegrees at wavelength λ, Mo is the mean residual weight of the protein, *c* is the protein concentration (mg/cm^3^), and *l* is the path length (cm).

### 2.5. Fluorescence Measurements

Fluorescence spectroscopy was performed with a PTI spectrofluorometer (Photon Technology International, Inc., Lawrenceville, NJ, USA) equipped with a single photon counting system for fluorescence intensity detection, dual fluorescence and absorbance channels using excitation and emission slits of 1 nm each. All measurements were performed at 25 °C in 50 mM Tris-HCl, 100 mM NaCl, pH 7.4 buffer. For tryptophan fluorescence measurements, the emission spectra were recorded in the wavelength range 310–450 nm after excitation at 295 nm to avoid tyrosine fluorescence. 

8-Anilinonaphthalene-1-sulfonate (ANS) fluorescence spectra of Prdx6 were collected from 400 to 600 nm after excitation at 360 nm. The protein concentration used for Trp and ANS fluorescence measurements was 1 µM. The ANS concentration in ANS fluorescence was 16 µM. All the measurements were done in micro quartz fluorescence cuvettes with pathlength of 0.3 cm (Sterna).

### 2.6. Thermal-Induced Denaturation

Heat-induced denaturation of Prdx6 was carried out in a Jasco-750 CD Spectropolarimeter equipped with a Peltier-type temperature controller with a heating rate of 1 °C/min, a scan rate providing adequate time for equilibration. Thermal denaturation was recorded following changes in [*θ*]_220_ from 20 °C to 80 °C at a rate of 1 °C/min in Jasco-750 CD Spectropolarimeter. A fused quartz cell with a pathlength of 1 cm was used. The protein concentration was 1 µM. The CD measured mean residual ellipticity at [*θ*]_220_ as a function of temperature is normalized at the start of the temperature scan. As shown earlier [22], each thermal denaturation curve was fitted to a two-state unfolding model after assuming a linear dependence of pre- and post-transition baselines on temperature [23,24]:

(1) $$y\;(T)\;=\frac{[(y_{N}+m_{N}T)+(y_{D}+m_{D}T)]\;\exp\;[-\Delta H_{m}/R(1/T-1/T_{m})]}{1+\exp[-\Delta H_{m}/R(1/T-1/T_{m})]}$$

*y*(*T*) is the observed mean residue ellipticity at a given temperature, *m*_*N*_ and *m*_*D*_ are slopes of the native and denatured baselines, while *y*_*N*_ and *y_D_* are the intercept of the native and denatured baselines, respectively. *T* is the temperature in kelvin, *T*_*m*_ is the melting temperature in kelvin and Δ*H*_*m*_ is the enthalpy change of denaturation at melting temperature and *R* is the universal gas constant. Curve fitting of the data points was done in Sigma Plot 13.0 software (Systat Software, Inc., San Jose, CA, USA) using Equation (1) in the dynamic fit.

### 2.7. Dynamic Light Scattering Measurements

The size distribution of the particles present in the protein sample was obtained using a Zetasizer Micro V/ZMV 2000 (Malvern, UK). The reduced and hyperoxidized samples were in the standard buffer, i.e., 50 mM Tris-HCl, 100 mM NaCl pH 7.4. The protein concentration was 2 mg/mL. All measurements were performed at 25 °C. Measurements were made at a fixed angle of 90° using an incident laser beam of 689 nm. Fifteen measurements were made with an acquisition time of 30 s for each sample at sensitivity of 10%. The data was analyzed using Zetasizer software provided by the manufacturer to get hydrodynamic diameters of the particles and the volume fraction of particles associated with that particular diameter. The hydrodynamic data obtained from this measurement was used to calculate the apparent molecular weight of reduced and hyperoxidized Prdx6.

### 2.8. Native PAGE

Reduced and hyperoxidized Prdx6 in the standard buffer, i.e., 50 mM Tris-HCl, 100 mM NaCl pH 7.4 were loaded in a continuous 7.5% polyacrylamide gel under non-reducing and non-denaturing condition. Proteins were electrophoresed using running buffer of 30 mM beta-alanine and 20 mM lactic acid (85–90%), pH 3.8 at a constant voltage (180 V) for 2 h with reverse polarity. The gel was run in the presence of two markers, bovine albumin (Mr 66.5 kDa, pI 4.7–4.9) and high molecular weight horse ferritin (Mr 450 kDa, pI 4.4) under non-reducing and non-denaturing conditions. Coomassie brilliant blue G-250 was used for the detection of the proteins on the gel.

### 2.9. Homology Modelling

The complete sequence of rat Prdx6 (consisting of 224 amino acids) was retrieved from UniProtKB database (accession number O35244). The best three templates, based on sequence identity (91.52%, 91.52%, 91.52%) and coverage (100%, 100% and 100%), of models with dimer option were generated from the selected templates (PDB codes: 1PRX, 5B6M and 5B6N), respectively. The reduced Cys47 was converted to hyperoxidized (Cys47-SH to Cys47-SO_3_H) form in Maestro [25]. To remove any steric clashes, energy minimization was performed using the GROMOS96 force field implemented in Swiss-Pdb Viewer [26].

PROCHECK [27] was used to verify the backbone conformation of the modeled structures, and the quality of the models was evaluated by qualitative model energy analysis (QMEAN) [28]. The QMEAN score gives the global score of the model and it ranges from 0 to 1, the higher the scores, the more reliable are the models. Further, the QMEAN Z-score provides an estimation of the “degrees of nativeness” of the structural features observed in a model and indicates model quality compared to experimental structures. The model having the best validation results was selected for further studies. 

### 2.10. Molecular Dynamics Simulation

Desmond [29] was used to perform MD simulations by implementing OPLS-AA 2005 force field [30,31] for reduced and hyperoxidized rat Prdx6 in its dimeric state. Protein Preparation wizard was used to prepare the protein structure in Schrodinger. TIP3P water model was selected to solvate the protein, the orthorhombic periodic boundary box was chosen to construct the required systems for subsequent MD simulations. Appropriate numbers of counter ions were added to the system to maintain charge neutrality. Further, the distance between the box and wall was set to be greater than 10 Å in order to avoid direct interaction with its own periodic image of the protein complex. Steepest descent method was selected to minimize the potential energies of the systems by applying a maximum of 5000 steps with a gradient threshold of 25 kcal mol^−1^ Å^−1^, followed by L-BFGS (low-memory Broyden-Fletcher-Goldfarb-Shanno quasi-Newtonian) minimizer until a convergence criterion of 1 kcal mol^−1^ Å^−1^ was achieved.

Default parameters were selected to equilibrate the pressure and temperature. Thereafter, the final MD simulation was done on the equilibrated systems for 50 ns at a constant temperature of 300 K, and constant pressure of 1 atm with a time step of 2 fs. To compute long-range electrostatic interactions, the particle-mesh Ewald method (PME) [32] was applied with a grid spacing of 0.8 Å. The van der Waals and short-range electrostatic interactions were smoothly truncated at 9.0 Å.

Root mean square deviation (RMSD), root mean square fluctuation (RMSF), radius of gyration, solvent accessible surface area (SASA), internal hydrogen bond contacts of the reduced and hyperoxidized Prdx6 and PCA were calculated using VMD tcl scripts.

## 3. Results and Discussion

**Figure 2:**
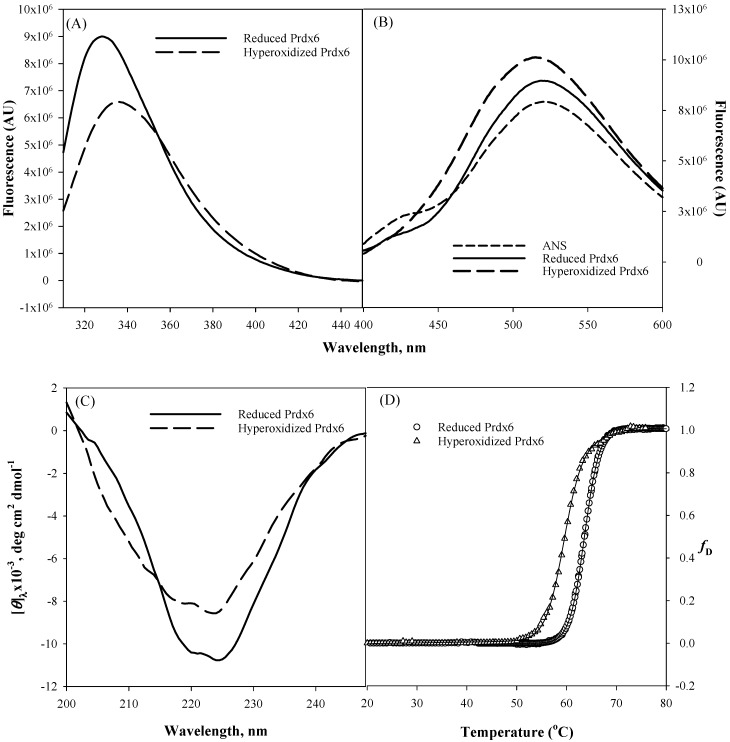
Tryptophan fluorescence (**A**), 8-Anilinonaphthalene-1-sulfonate (ANS) fluorescence (**B**), Far-Ultraviolet (UV) Circular Dichroism CD (**C**) measurements, and normalized thermal-induced denaturation curves (**D**) of reduced Prdx6 and hyperoxidized Prdx6. All measurements were done at pH 7.4 (50 mM Tris-HCl, 100 mM NaCl) and at 25 °C. Thermal denaturation was recorded following changes in [*θ*]_220_ from 20 °C to 80 °C at a rate of 1 °C/min. All spectra are the mean of three independent experiments.

It is universally known that the ultimate folded native state conformation, not the primary structure of a protein determines the functional capacity and efficiency of a protein. Therefore, for multifunctional proteins (such as Prdx6, aconitase, activating transcription factor 2, ERK2, MAP kinase, glutamate racemase, etc.) usage of native state conformational change as a mechanism to switch between two different activities seems very common [22,33]. To comprehend structural alterations as a possibility for upregulation of aiPLA2 activity due to the hyperoxidation of peroxidatic cysteine residue of Prdx6 [19], we first did the in vitro comparison of the secondary and tertiary structure of hyperoxidized and reduced Prdx6 via various spectroscopic methods (Figure 2). Tryptophan fluorescence emission is a very sensitive tool for measuring the polarity of the local environment of tryptophan residues [34], and hence, mostly used as a diagnostic probe for analyzing conformational state of proteins [35]. Prdx6 has three tryptophan (Trp) residues, Trp33, Trp82 and Trp181. The solvent exposure of these Trp residues under different redox conditions is assessed by measuring tryptophan fluorescence of reduced and hyperoxidized Prdx6. As shown in Figure 2A, a decrease in fluorescence intensity and red shift from 328 nm to 334 nm of the hyperoxidized Prdx6 in comparison to that of reduced Prdx6 clearly reveals that Trp residues of hyperoxidized Prdx6 (relative to reduced protein) have been shifted to a more polar solvent environment.

To confirm the likelihood of greater exposition of hydrophobic amino acids in hyperoxidized Prdx6 (as suggested by the Trp fluorescence results), we investigated the ANS binding behaviour of reduced and hyperoxidized Prdx6. The fluorescent hydrophobic probe 8-Anilinonaphthalene-1-sulfonate (ANS) is usually used to detect the accessible hydrophobic surfaces on proteins [36,37]. In aqueous solvent, the quantum yield of the ANS probe is very weak, which on binding to a hydrophobic surface increases several fold [38]. As seen in Figure 2B, ANS fluorescence measurement in aqueous solvent shows an emission maximum at ~520 nm, and at 519 nm and 515 nm with reduced and hyperoxidized Prdx6, respectively. And there is increase in fluorescence intensity of the hyperoxidized Prdx6 in comparison to that of the aqueous solvent and reduced Prdx6. The blue shift in emission maxima and increase in the fluorescence intensity of ANS fluorescence with hyperoxidized Prdx6 as compared to the aqueous solvent is a clear signature for ANS binding, and indicates the exposure of hydrophobic amino acids to the solvent in the hyperoxidized Prdx6. Such exposure of hydrophobic amino acids in oxidized Prdx6, suggests conformational alterations with opening of the hydrophobic core.

We further analyzed whether the disparity in the hydrophobic core’s packing within reduced and hyperoxidized Prdx6 is limited to only tertiary alterations or also has its root in the changes at secondary structure level. The far-UV region of the circular dichroism (CD) spectrum monitors the changes in the secondary structure of protein [39,40]. Here we evaluated the effect of redox state on the secondary structure of Prdx6 by performing CD measurements with reduced and hyperoxidized Prdx6 in the far-UV region. The mean residual ellipticity at *θ*_222_, the measure of secondary structure content of Prdx6, is shown in Table 1. Figure 2C and Table 1 show the far-UV CD spectra of both proteins, and the decrease in secondary structure content of hyperoxidized Prdx6 (in comparison with reduced Prdx6) indicates the influence of the redox state of Prdx6 for the functional switching of the protein by altering its secondary as well as tertiary structure. Taken together, the results indicate that there is a conformational difference between the reduced and hyperoxidized protein. However, we do not rule out the possibility that oligomer formation may also contribute to the observed spectral properties of the hyperoxidized protein.

We were further interested to see the impact of oxidation mediated conformational change on the thermodynamic stability of Prdx6. To investigate this, we measured the thermodynamic stability of reduced and hyperoxidized Prdx6 via an optical method, i.e., by monitoring the changes in molar ellipticity at 220 nm over the temperature range of 20–80 °C (Figure 2D). The transition curve obtained from the thermal-induced denaturation was analyzed using a two-state unfolding model, given above in Equation (1). The analysis gave the change in melting temperature (*T*_*m*_) and change in enthalpy (Δ*H*_*m*_) at the melting temperature of Prdx6, as shown in Table 1. As seen in Figure 2D and Table 1, the hyperoxidized Prdx6 shows a decrease of 4 °C in its *T*_*m*_ as compared to that of reduced Prdx6. This decreased stability shows the conformational change of the native state of Prdx6 upon hyperoxidation, which may lead to changes in its activity under physiological condition.

**Figure 3:**
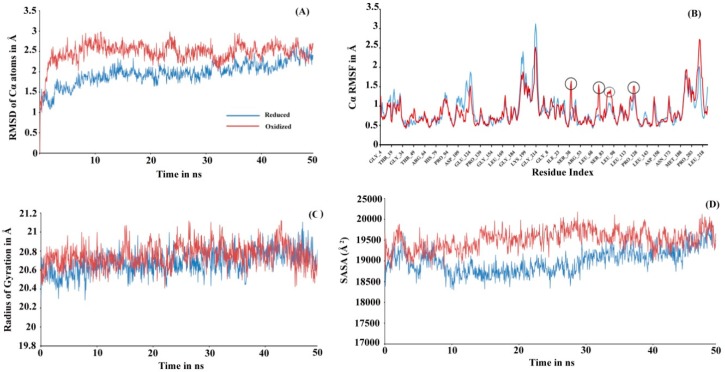
(**A**) RMSD, (**B**) RMSF, (**C**) Radius of gyration and **(D**) SASA plot of reduced (blue) and hyperoxidized (red) Prdx6 Cα atoms along the 50 ns simulation period. RMSD: Root mean square deviation; RMSF: Root mean square fluctuation; SASA: Solvent accessible surface area.

To investigate the actual molecular level differences in the tertiary interaction, we performed molecular dynamic (MD) simulations for both the proteins (see Figure 3). The initial 3D structure models used while initiating simulation were run at Prdx6 homology models with their peroxidatic cysteine either reduced (Cys47-SH) or oxidized (Cys47-SO_3_H). The structure validation for the modeled rPrdx6 protein was done via PROCHECK (Ramachandran plot generator, Figure S1) and QMEAN (Figure S2) in-silico tools demonstrated the predicted model had good correlation with the experimental structures and were acceptable for further use. In MD simulation, root mean square deviation (RMSD) changes in Cα atoms of the reduced Prdx6 showed a slow increment during the simulation and finally converged at the end of the simulation around 2.5 Å. At the same time, hyperoxidized Prdx6 showed fluctuations at the start, reached 3 Å by 200 ns, and ultimately converging at 2.5 Å, indicating more fluctuation in oxidized Prdx6 compared to reduced Prdx6 (Figure 3A). The root mean square fluctuation (RMSF) analysis is an important criterion to measure the stability of the protein under study. As seen in Figure 3B, the hyperoxidized Prdx6 displayed more fluctuations in 4 regions (in comparison to reduced Prdx6) localized in the conserved thioredoxin fold. It must be noted that apart from these 4 peaks, the fluctuations showed by side chains of other amino acids were almost similar, and highly flexible regions seems to mostly lie in the loops. In terms of conformational analysis, the radius of gyration is described as the moment of inertia of the group of atoms from their center of mass. As shown in Figure 3C, we observed, that the hyperoxidized Prdx6 showed a higher gyration radius than that of the reduced Prdx6 throughout the simulation run. Further details on the structural packing of the proteins were assessed by measuring SASA. SASA provides insight about the compactness of the hydrophobic core, which is an important factor for determining protein stability. As illustrated in Figure 3D, the SASA profile of the hyperoxidized Prdx6 simulation run exhibited a higher SASA value than that of reduced Prdx6, which could explain the differences in polarity of the local environment of tryptophan residues in both forms of the protein. However, it seems that the hyperoxidation of Prdx6 did not affect the overall dynamics or stability of the protein, indeed, it somehow disrupted the packing of the hydrophobic core, leading to solvent exposition of hydrophobic amino acids as evidenced by the higher gyration radii, SASA profile, and ANS fluorescence of hyperoxidized Prdx6 as compared to reduced Prdx6. 

**Figure 4:**
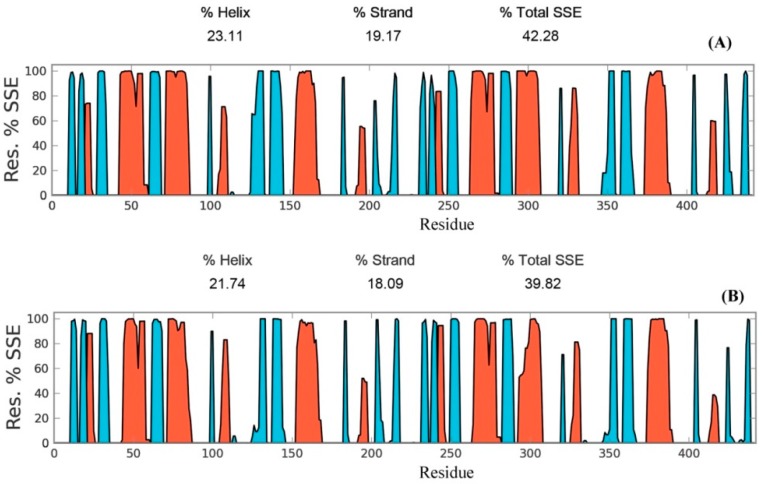
Secondary structure elements evolution of reduced (**A**) and hyperoxidized (**B**) Prdx6 during the simulation period. Helices are shown in red and strands are shown in cyan.

**Figure 5:**
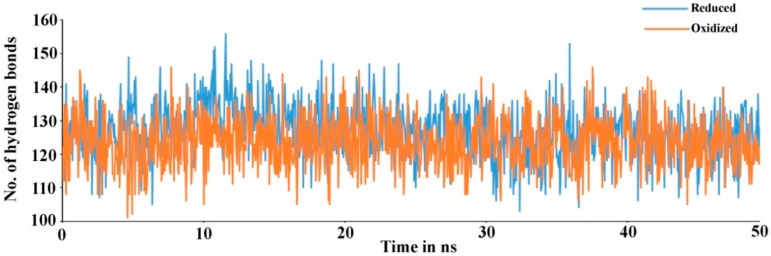
Total number of hydrogen bond found in reduced (blue) and hyperoxidized (orange) Prdx6 during the simulation period.

Secondary structure elements, such as alpha-helices and beta-sheets were also monitored throughout the simulation period (see Figure 4). There are 23.11% helix and 19.17% beta-sheets in reduced Prdx6, while the hyperoxidized Prdx6 has 21.74% helix and 18.09% beta-strands, thereby, the total percentage difference in secondary structure elements in both proteins is only 2%. To assess the contribution of non-covalent interaction on the flexibility of Prdx6, hydrogen bond formation during the simulation period was monitored. For each trajectory frame, total hydrogen bonds present in the protein were calculated. The hydrogen bond analysis revealed a decrease in the total number of hydrogen bonds in hyperoxidized Prdx6 (Figure 5) as compared to reduced Prdx6 during the simulation period. The average hydrogen bonds throughout the simulation for hyperoxidized Prdx6 was calculated to be 123.515 while for the reduced protein it was 126.823. However, since this loss does not seem to affect the internal dynamics of the protein (as indicated by RMSD and RMSF plots), the reason for up-regulation of aiPLA_2_ activity in hyperoxidized Prdx6 remains ambiguous.

**Figure 6:**
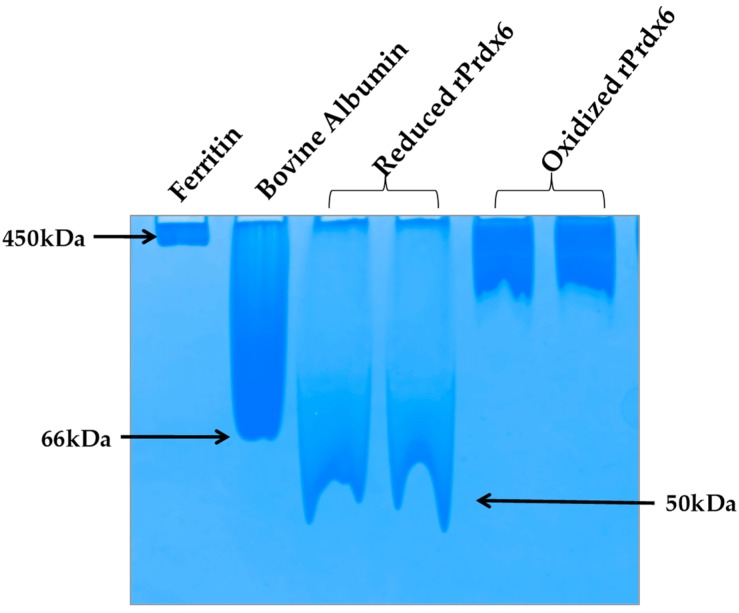
Native-PAGE.Reduced and hyperoxidized Pdrx6s along with bovine serum albumin and horse ferritin as a marker under non-reducing and non-denaturing conditions in the buffer 150 mM beta- alanine and 100 mM lactic acid (85–90%), pH 3.8.

Prdxs are inherently oligomeric proteins whose multimeric status is known to be influenced by the oxidation states of the peroxidatic cysteine. For instance, hyperoxidation of peroxidatic cysteine to a Cys–SO_2_H and Cys–SO_3_H under oxidative stress has been reported to alter the quaternary structure of a typical 2-Cys Prdx from a dimer into higher oligomers, thus, forming molecular complexes that led to inactivation of its peroxidase activity [41,42]. In fact, in a homolog of Prdx, this structural change from low molecular weight multimer to a high molecular weight oligomeric complex, has been observed to cause a functional change from peroxidase to molecular chaperone [43]. In addition, subunit associations are often preceded by conformational transitions that cause the exposition of hydrophobic amino acids on the surface for stronger interactions at the oligomeric interface. Taking all this into consideration, it is highly probable that hyperoxidation of Prdx6 causes a similar effect (as that of other Prdxs) on the quaternary structure of the enzyme. To analyze the native state oligomeric composition of Prdx6, the native-PAGE of reduced and hyperoxidized Prdx6 was performed (see Figure 6). It must be noted here that, the crystallographic studies as well as equilibrium sedimentation and DuoLink analyses have shown Prdx6 in reduced (Cys47-SH) and sulfinic forms (Cys47-SO_2_H) to exist as dimers [44,45,46]. In native PAGE gel we also observed the dimeric nature of reduced Prdx6, as indicated by the protein band (monomer molecular weight, M_r_: 25 KDa), which is close to that of serum albumin (M_r_: 64kDa). However, the hyperoxidized Prdx6 showed a thick band similar to that of horse ferritin, having M_r_ of 450 kDa.

This varied mobility of both proteins on a native PAGE gel, indicates that hyperoxidized Prdx6 exists in a higher oligomer state. These observations were confirmed by the dynamic light scattering (DLS) measurements at physiological pH 7.4 (See Table 1). The reduced Prdx6 was found to have a hydrodynamic radius of 5.36 nm (corresponding to that of a protein with M_r_ 50 KDa) while the hyperoxidized Prdx6 had a hydrodynamic radius of 36.19 nm.; the apparent molecular weight calculated from the hydrodynamic radii of reduced and hyperoxidized Prdx6 are shown in Table 1. Thus, we concluded that the quaternary state of reduced and hyperoxidized Prdx6 is different. This change in the quaternary structure of the proteins is preceded by the conformational change shown by the above biophysical studies (CD and fluorescence) for its subunit association during oligomerization [43,47]. The exposure of hydrophobic residues in hyperoxidized Prdx6 (detected by ANS fluorescence) is necessary for non-covalent interaction among subunits in high oligomers. However, the lesser change in the internal dynamics of the protein (as indicated by RMSD and RMSF plots) might be because MD simulation of hyperoxidized Prdx6 was performed in its dimeric state.

During the redox cycle, the hydrophobic residues (Leu 145, Ile 147, Leu 148 and Tyr 149) between β-strands (βb7 from each monomer) in Prdx6 play a significant role on the stabilization of Prdx6-Prdx6 homodimerization and Prdx6-πGST heterodimerization (an important step on its peroxidase activity) [44]. Our finding that the formation of higher oligomer of Prdx6 on the hyperoxidation may not allow the formation of Prdx6-πGST hetero-dimer to recycle the hyperoxidized Prdx6, leading to loss of its peroxidase activity but it might overexpose the catalytic triad for aiPLA2 activity located at the protein surface, inducing the upregulation of its phosphopholipase activity, which has previously been shown in [19]. Therefore, the results lead us to propose that the change in the oligomeric state of Prdx6 due to hyperoxidation might induce the switching of its peroxidase activity to aiPLA2 activity. 

## 4. Conclusions

In summary, our study demonstrates that post-translational modifications (like hyperoxidation) cause the alteration of reduced Prdx6 dimer to a multimeric status. The formation of a high order oligomer is consequently due to a decrease in protein stability as a result of the opening of some of the hydrophobic groups to the polar solvent. In future, it will be important to further identify the properties of this oligomeric protein and the physiological significance thereof.

## Figures and Tables

**Table 1:** Conformational and thermodynamic parameters of reduced and hyperoxidized Prdx6.

Prdx6 Species	[*θ*]_222_ × 10^−3^, Mdeg cm^2^dmol^−1^	Δ*H*_*m*_, kcal mol^−1^	*T*_*m*_, (°C)	Hydrodynamic Radii, *R_h_* (nm)	Apparent Molecular Weight (kD)
Reduced Prdx6	10.5 ± 0.02	634.10 ± 45.58	63.6 ± 0.5	5.36 ± 0.14	50.0
Hyperoxidized Prdx6	8.4 ± 0.05	499.65 ± 64.57	59.4 ± 0.2	36.19 ± 0.23	337.6

Mean ± S. D. from three independent experiments.

## Supplementary Materials

The following are available online at http://www.mdpi.com/2076-3921/8/2/33/s1, Figure S1: Ramachandran plot of rat Prdx6 model generated using Procheck. Figure S2: Graphical representation of the absolute quality of rat Prdx6 model evaluated by QMEAN Z-score.

## References

[1] Wood Z.A., Schroder E., Robin Harris J., Poole L.B. (2003). Structure, mechanism and regulation of peroxiredoxins. Trends Biochem. Sci..

[2] Valko M., Leibfritz D., Moncol J., Cronin M.T., Mazur M., Telser J. (2007). Free radicals and antioxidants in normal physiological functions and human disease. Int. J. Biochem. Cell Biol..

[3] Rhee S.G., Kang S.W., Chang T.S., Jeong W., Kim K. (2001). Peroxiredoxin, a novel family of peroxidases. IUBMB Life.

[4] Halliwell B. (1996). Antioxidants in human health and disease. Annu Rev. Nutr..

[5] Sies H. (1993). Strategies of antioxidant defense. Eur. J. Biochem..

[6] Mizohata E., Sakai H., Fusatomi E., Terada T., Murayama K., Shirouzu M., Yokoyama S. (2005). Crystal structure of an archaeal peroxiredoxin from the aerobic hyperthermophilic crenarchaeon Aeropyrum pernix K1. J. Mol. Biol..

[7] Rhee S.G., Chae H.Z., Kim K. (2005). Peroxiredoxins: A historical overview and speculative preview of novel mechanisms and emerging concepts in cell signaling. Free Radic. Biol. Med..

[8] Perkins A., Nelson K.J., Parsonage D., Poole L.B., Karplus P.A. (2015). Peroxiredoxins: Guardians against oxidative stress and modulators of peroxide signaling. Trends Biochem. Sci..

[9] Fujii J., Ikeda Y. (2002). Advances in our understanding of peroxiredoxin, a multifunctional, mammalian redox protein. Redox Rep..

[10] Atkinson H.J., Babbitt P.C. (2009). An atlas of the thioredoxin fold class reveals the complexity of function-enabling adaptations. PLoS Comput. Biol..

[11] Seo M.S., Kang S.W., Kim K., Baines I.C., Lee T.H., Rhee S.G. (2000). Identification of a new type of mammalian peroxiredoxin that forms an intramolecular disulfide as a reaction intermediate. J. Biol. Chem..

[12] Manevich Y., Feinstein S.I., Fisher A.B. (2004). Activation of the antioxidant enzyme 1-CYS peroxiredoxin requires glutathionylation mediated by heterodimerization with pi GST. Proc. Natl. Acad. Sci. USA.

[13] Choi H.J., Kang S.W., Yang C.H., Rhee S.G., Ryu S.E. (1998). Crystallization and preliminary X-ray studies of hORF6, a novel human antioxidant enzyme. Acta Crystallogr. D Biol. Crystallogr..

[14] Fisher A.B. (2016). Peroxiredoxin 6 in the repair of peroxidized cell membranes and cell signaling. Arch. Biochem. Biophys..

[15] Manevich Y., Shuvaeva T., Dodia C., Kazi A., Feinstein S.I., Fisher A.B. (2009). Binding of peroxiredoxin 6 to substrate determines differential phospholipid hydroperoxide peroxidase and phospholipase A(2) activities. Arch. Biochem. Biophys..

[16] Akiba S., Dodia C., Chen X., Fisher A.B. (1998). Characterization of acidic Ca^2+^-independent phospholipase A2 of bovine lung. Comp. Biochem. Physiol. B Biochem. Mol. Biol..

[17] Li H., Benipal B., Zhou S., Dodia C., Chatterjee S., Tao J.Q., Sorokina E.M., Raabe T., Feinstein S.I., Fisher A.B. (2015). Critical role of peroxiredoxin 6 in the repair of peroxidized cell membranes following oxidative stress. Free Radic. Biol. Med..

[18] Fisher A.B. (2018). The phospholipase A2 activity of peroxiredoxin 6. J. Lipid Res..

[19] Kim S.Y., Jo H.Y., Kim M.H., Cha Y.Y., Choi S.W., Shim J.H., Kim T.J., Lee K.Y. (2008). H2O2-dependent hyperoxidation of peroxiredoxin 6 (Prdx6) plays a role in cellular toxicity via up-regulation of iPLA2 activity. J. Biol. Chem..

[20] Chong S., Mersha F.B., Comb D.G., Scott M.E., Landry D., Vence L.M., Perler F.B., Benner J., Kucera R.B., Hirvonen C.A. (1997). Single-column purification of free recombinant proteins using a self-cleavable affinity tag derived from a protein splicing element. Gene.

[21] Moll J.R., Ruvinov S.B., Pastan I., Vinson C. (2001). Designed heterodimerizing leucine zippers with a ranger of pIs and stabilities up to 10(-15) M. Protein Sci..

[22] Rahaman H., Zhou S., Dodia C., Feinstein S.I., Huang S., Speicher D., Fisher A.B. (2012). Increased phospholipase A2 activity with phosphorylation of peroxiredoxin 6 requires a conformational change in the protein. Biochemistry.

[23] Santoro M.M., Bolen D.W. (1988). Unfolding free energy changes determined by the linear extrapolation method. 1. Unfolding of phenylmethanesulfonyl alpha-chymotrypsin using different denaturants. Biochemistry.

[24] Khan A., Das M.K., Das U., Rahaman M.H., Hassan M.I., Srinivasan A., Singh T.P., Ahmad F. (2009). A single mutation induces molten globule formation and a drastic destabilization of wild-type cytochrome c at pH 6.0. J. Biol. Inorg. Chem. JBIC.

[25] Maestro (2018). Schrödinger Release.

[26] Guex N., Peitsch M.C. (1997). SWISS-MODEL and the Swiss-PdbViewer: An environment for comparative protein modeling. Electrophoresis.

[27] Laskowski R.A., Moss D.S., Thornton J.M. (1993). Main-chain bond lengths and bond angles in protein structures. J. Mol. Biol..

[28] Benkert P., Tosatto S.C., Schomburg D. (2008). QMEAN: A comprehensive scoring function for model quality assessment. Proteins.

[29] Bowers K.J., Chow D.E., Xu H., Dror R.O., Eastwood M.P., Gregerse B.A., Klepeis J.L., Kolossvary I., Moraes M.A., Sacerdoti F.D. Shaw Scalable algorithms for molecular dynamics simulations on commodity clusters. Proceedings of the 2006 ACM/IEEE Conference on Supercomputing.

[30] Kaminski G.A., Friesner R.A., Tirado-Rives J., Jorgensen W.L. (2001). Evaluation and Reparametrization of the OPLS-AA Force Field for Proteins via Comparison with Accurate Quantum Chemical Calculations on Peptides. J. Phys. Chem. B.

[31] Jorgensen W.L., Maxwell D.S., Tirado-Rives J. (1996). Development and Testing of the OPLS All-Atom Force Field on Conformational Energetics and Properties of Organic Liquids. J. Am. Chem. Soc..

[32] Essmann U., Perera L., Berkowitz M.L., Darden T., Lee H., Pedersen L.G. (1995). A Smooth Particle Mesh Ewald Method. J. Chem. Phys..

[33] Jeffery C.J. (2016). Protein species and moonlighting proteins: Very small changes in a protein’s covalent structure can change its biochemical function. J. Proteom..

[34] Lakowicz J.R. (1999). Principles of Fluorescence Spectroscopy.

[35] Vivian J.T., Callis P.R. (2001). Mechanisms of tryptophan fluorescence shifts in proteins. Biophys. J..

[36] Semisotnov G.V., Rodionova N.A., Razgulyaev O.I., Uversky V.N., Gripas A.F., Gilmanshin R.I. (1991). Gilmanshin, Study of the “molten globule” intermediate state in protein folding by a hydrophobic fluorescent probe. Biopolymers.

[37] Stryer L. (1965). The interaction of a naphthalene dye with apomyoglobin and apohemoglobin. A fluorescent probe of non-polar binding sites. J. Mol. Biol..

[38] Rosen C.G., Weber G. (1969). Dimer formation from 1-amino-8-naphthalenesulfonate catalyzed by bovine serum albumin. A new fluorescent molecule with exceptional binding properties. Biochemistry.

[39] Yang J.T., Wu C.S., Martinez H.M. (1986). Calculation of protein conformation from circular dichroism. Methods Enzymol..

[40] Kelly S.M., Jess T.J., Price N.C. (2005). How to study proteins by circular dichroism. Biochim. Biophys. Acta.

[41] Schroder E., Littlechild J.A., Lebedev A.A., Errington N., Vagin A.A., Isupov M.N. (2000). Crystal structure of decameric 2-Cys peroxiredoxin from human erythrocytes at 1.7 A resolution. Structure.

[42] Jang H.H., Lee K.O., Chi Y.H., Jung B.G., Park S.K., Park J.H., Lee J.R., Lee S.S., Moon J.C., Yun J.W. (2004). Two enzymes in one; two yeast peroxiredoxins display oxidative stress-dependent switching from a peroxidase to a molecular chaperone function. Cell.

[43] Wood Z.A., Poole L.B., Hantgan R.R., Karplus P.A. (2002). Dimers to doughnuts: Redox-sensitive oligomerization of 2-cysteine peroxiredoxins. Biochemistry.

[44] Zhou S., Sorokina E.M., Harper S., Li H., Ralat L., Dodia C., Speicher D.W., Feinstein S.I., Fisher A.B. (2016). Peroxiredoxin 6 homodimerization and heterodimerization with glutathione S-transferase pi are required for its peroxidase but not phospholipase A2 activity. Free Radic. Biol. Med..

[45] Choi H.J., Kang S.W., Yang C.H., Rhee S.G., Ryu S.E. (1998). Crystal structure of a novel human peroxidase enzyme at 2.0 A resolution. Nat. Struct. Biol..

[46] Lee W., Kim K.H., Kim E.E. (2016). Crystal structures of human peroxiredoxin 6 in different oxidation states. Biochem. Biophys. Res. Commun..

[47] Rhee S.G., Woo H.A. (2011). Multiple functions of peroxiredoxins: Peroxidases, sensors and regulators of the intracellular messenger H_2_O_2_, and protein chaperones. Antioxid. Redox Signal..

