# Supplementary material for: Hyperoxidation of Peroxiredoxin 6 Induces Alteration from Dimeric to Oligomeric State
Source: Antioxidants (Basel). 2019 Feb 2;8(2):33. doi: 10.3390/antiox8020033 (PMC6406459; doi:10.3390/antiox8020033)
Supplement: Supplementary file 1 [file antioxidants-08-00033-s001.pdf]

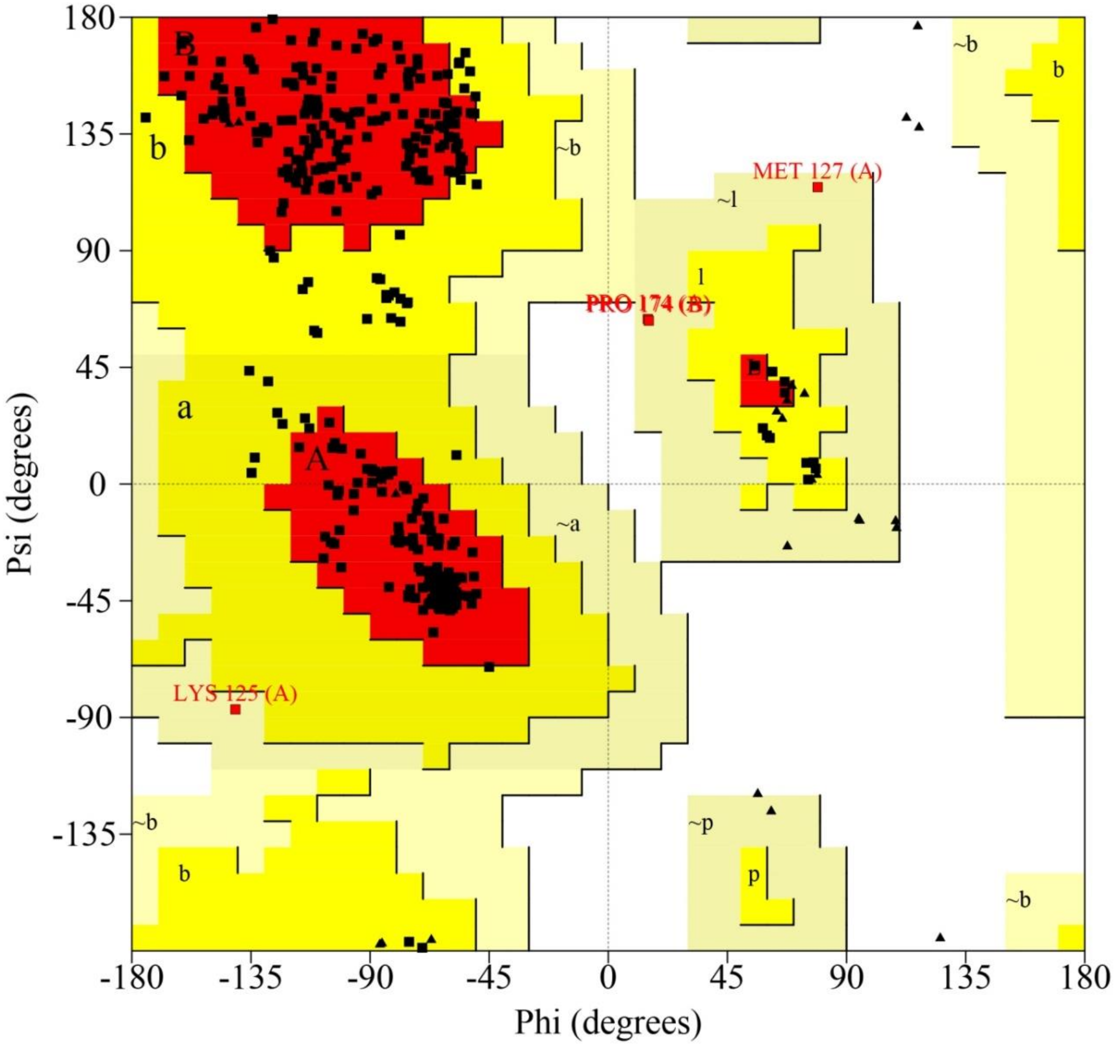

Plot statistics

Residues in most favoured regions [A,B,L]	339	90.4%
Residues in additional allowed regions [a,b,l,p]	34	9.1%
Residues in generously allowed regions [~a,~b,~l,~p]	2	0.5%
Residues in disallowed regions	0	0.0%
	----	------
Number of non-glycine and non-proline residues	375	100.0%
Number of end-residues (excl. Gly and Pro)	1	
Number of glycine residues (shown as triangles)	28	
Number of proline residues	39	

Total number of residues	443	

Figure S1: Ramachandran plot of rat Prdx 6 model generated using Procheck. Different areas of the plot are represented with different colors; beige, yellow, brown and red indicating disallowed, generously allowed, additional allowed and most favored regions, respectively.

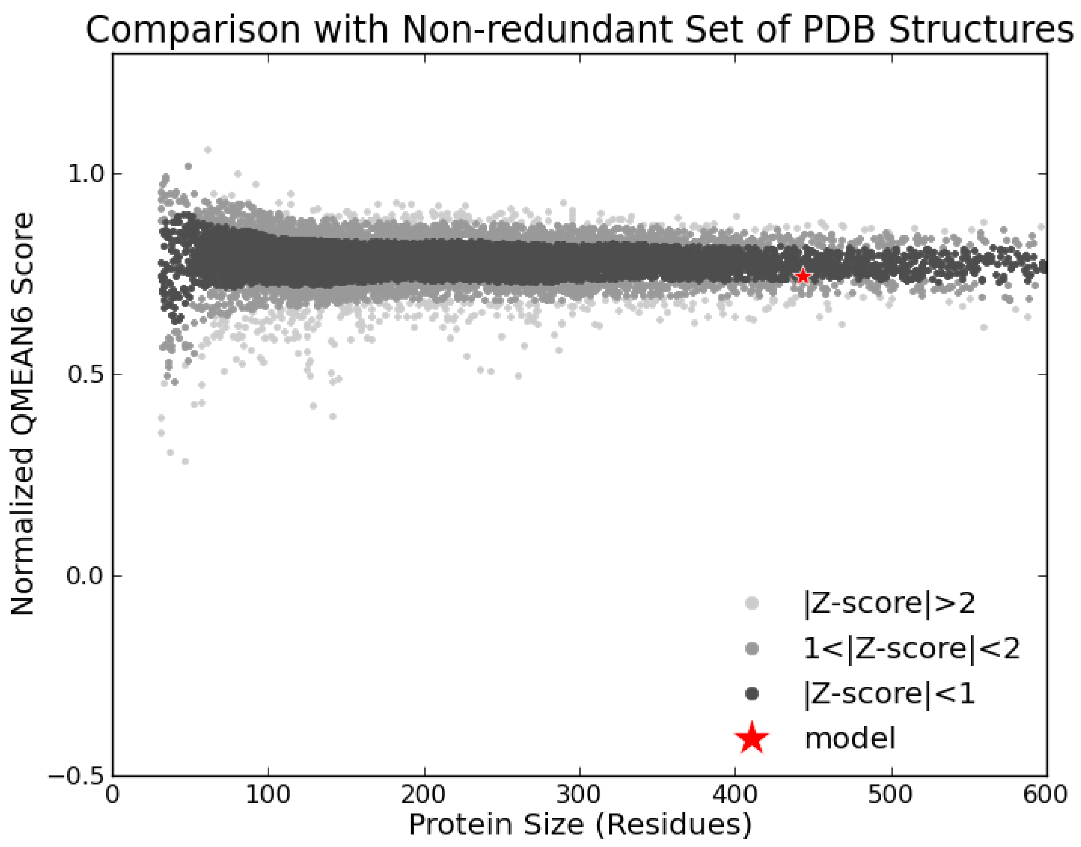

Figure S2: Graphical representation of the absolute quality of rat Prdx6 model evaluated by QMEAN Z-score. The red mark indicates the position of Prdx6 model.
